# Cerebral hierarchies: predictive processing, precision and the pulvinar

**DOI:** 10.1098/rstb.2014.0169

**Published:** 2015-05-19

**Authors:** Ryota Kanai, Yutaka Komura, Stewart Shipp, Karl Friston

**Affiliations:** 1 School of Psychology, Sackler Centre for Consciousness Science, University of Sussex, Brighton BN1 9QH, UK; 2 Precursory Research for Embryonic Science and Technology, Japan Science and Technology Agency, Saitama 332-0012, Japan; 3 Systems Neuroscience, Human Technology Research Institute, National Institute of Advanced Industrial Science and Technology, Tsukuba 305-8568, Japan; 4 UCL Institute of Ophthalmology, London EC1V 9EL, UK; 5 Wellcome Trust Centre for Neuroimaging, Institute of Neurology, UCL, London WC1 3BG, UK

**Keywords:** neuronal computational, attention, predictive coding, precision, neuromodulation, pulvinar

## Abstract

This paper considers neuronal architectures from a computational perspective and asks what aspects of neuroanatomy and neurophysiology can be disclosed by the nature of neuronal computations? In particular, we extend current formulations of the brain as an organ of inference—based upon hierarchical predictive coding—and consider how these inferences are orchestrated. In other words, what would the brain require to dynamically coordinate and contextualize its message passing to optimize its computational goals? The answer that emerges rests on the delicate (modulatory) gain control of neuronal populations that select and coordinate (prediction error) signals that ascend cortical hierarchies. This is important because it speaks to a hierarchical anatomy of extrinsic (between region) connections that form two distinct classes, namely a class of driving (first-order) connections that are concerned with encoding the content of neuronal representations and a class of modulatory (second-order) connections that establish context—in the form of the salience or *precision* ascribed to content. We explore the implications of this distinction from a formal perspective (using simulations of feature–ground segregation) and consider the neurobiological substrates of the ensuing precision-engineered dynamics, with a special focus on the pulvinar and attention.

## 1. Introduction

There are many fascinating aspects of cerebral cartography that have been disclosed over the past centuries and—presumably—many more that have yet to reveal themselves. In this paper, we focus on one particular aspect, namely the imperatives for the coordination of message passing in the brain—and what those imperatives mandate in terms of cortical (and subcortical) anatomy. We base our treatment on the assumption that cerebral cartography is an anatomy of inference. In other words, the brain is a statistical organ predicting worldly states that generate its sensory inputs. In particular, we focus on predictive coding as a (biologically plausible) implementation of hierarchical inference in the brain and see how far this takes us in understanding the orchestration and contextualization of neuronal dynamics.

In what follows, we briefly review predictive coding with a special focus on how the brain encodes irreducible uncertainty inherent in the sensory evidence it samples [1,2]. It transpires that—under predictive coding—this uncertainty or relative confidence in sensory (and extrasensory) information can be succinctly encoded by the gain of certain neuronal populations that pass information from one hierarchal cortical level to the next [2,3]. This immediately brings us into the realm of cortical gain control and neuromodulation—that may be closely tied to synchronous gain and the (oscillatory) dynamics associated with binding, attention and dynamic coordination [4,5]. We then consider the computational anatomy implied by encoding the confidence or *precision* of ascending neuronal signals that is illustrated with a simple problem, namely figure–ground segregation in the visual hierarchy. We then turn to the neurobiology of cortical gain control, using the pulvinar as a prime example of a subcortical structure that has all the equipment necessary for contextualizing hierarchical inference in cortical hierarchies.

## 2. The Bayesian brain

**Figure 1.**
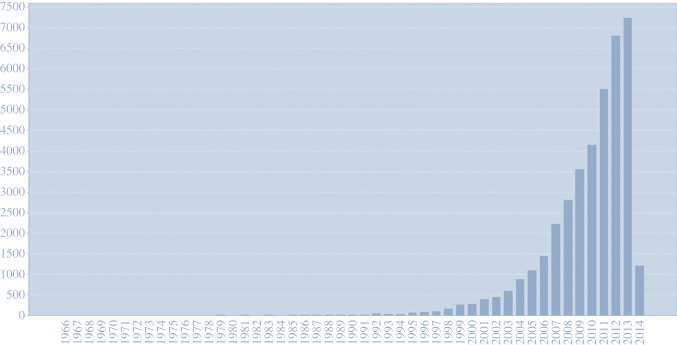
Citations per year, from 1966 to 2014, when searching for TOPIC: (Bayesian) AND TOPIC: (brain) in Web of Science. (Online version in colour.)

Recent advances in theoretical neuroscience have inspired a paradigm shift in cognitive neuroscience (figure 1). This shift is away from the brain as a passive filter of sensations towards a view of the brain as a statistical organ that generates hypotheses or fantasies which are tested against sensory evidence [6]. In this formulation, the brain is, literally, a fantastic organ (fantastic: from Greek *phantastikos*, the ability to create mental images, from *phantazesthai*). This perspective can be traced back to Helmholtz and the notion of unconscious inference [7]. This notion has been generalized to cover deep or hierarchical Bayesian inference—about the causes of our sensations—and how these inferences induce beliefs, movement and behaviour [8–12].

### (a) Predictive coding and the Bayesian brain

Modern formulations of Helmholtz's notion are now among the most popular explanations for neuronal message passing and are usually considered under the Bayesian brain hypothesis as predictive coding [12–15]. There is now considerable (circumstantial) anatomical and physiological evidence for predictive coding in the brain [15,16]. See Bastos *et al.* [17] for a review of canonical microcircuits and hierarchical predictive coding in perception and Adams *et al.* and Shipp *et al.* [18,19] for an equivalent treatment of the motor system. In these schemes, neuronal representations in higher levels of cortical hierarchies generate predictions of representations in lower levels. These top-down predictions are compared with representations at the lower level to form a prediction error (associated with the activity of superficial pyramidal cells). The ensuing mismatch signal is passed back up the hierarchy, to update higher representations (associated with the activity of deep pyramidal cells). This recursive exchange of signals suppresses prediction error at each and every level to provide a hierarchical explanation for sensory inputs that enter at the lowest (sensory) level. In computational terms, neuronal activity encodes beliefs or probability distributions over states in the world that cause sensations (e.g. my visual sensations are caused by a *face*). The simplest encoding corresponds to representing the belief with the expected value of a (hidden) cause or *expectation*. These causes are referred to as *hidden* because they have to be inferred from their sensory consequences.

**Figure 2.**
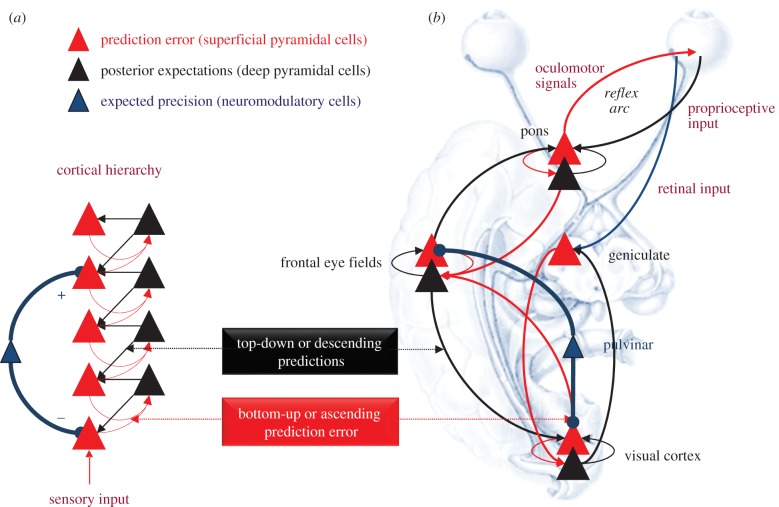
Summary of hierarchical message passing in predictive coding. Neuronal activity encodes expectations about the causes of sensory input, where these expectations minimize prediction error. Prediction error is the difference between (ascending) sensory input and (descending) predictions of that input. This minimization rests upon recurrent neuronal interactions among different levels of the cortical hierarchy. The available evidence suggests that superficial pyramidal cells (red triangles) compare the expectations (at each level) with top-down predictions from deep pyramidal cells (black triangles) of higher levels: see Bastos *et al.* [17] for a review of this evidence. (*a*) This schematic shows a simple cortical hierarchy with ascending prediction errors and descending predictions. We have included neuromodulatory gating or gain control (blue) of superficial pyramidal cells that determines their relative influence on deep pyramidal cells encoding expectations. (*b*) This provides a schematic example in the visual system: it shows the putative cells of origin of ascending or forward connections that convey prediction errors (red arrows) and descending or backward connections (black arrows) that construct predictions. The prediction errors are weighted by their expected precision—which we have associated with projections from the pulvinar. In this example, the frontal eye fields send predictions to primary visual cortex, which projects to the lateral geniculate body. However, the frontal eye fields also send proprioceptive predictions to pontine nuclei, which are passed to the oculomotor system to cause movement through classical reflexes. Every top-down prediction is reciprocated with a bottom-up prediction error to ensure predictions are constrained by sensory information.

In summary, predictive coding represents a biologically plausible scheme for updating beliefs (or expectations) about the world using sensory samples (figure 2). In this setting, neuroanatomy and neurophysiology can be regarded as a biological embodiment of how sensory signals are generated; for example, a smiling face generates luminance surfaces that generate textures and edges and so on, down to retinal input. This form of hierarchical inference explains a remarkable number of anatomical and physiological facts as documented elsewhere [15,17,18]. In brief, it explains the hierarchical nature of cortical cartography; the prevalence of backward connections and many of the functional and structural asymmetries in the extrinsic connections that link hierarchical levels. These asymmetries include the laminar specificity of forward and backward connections, the prevalence of nonlinear or modulatory backward connections (that embody interactions and nonlinearities inherent in the generation of sensory signals) and their spectral characteristics—with fast (e.g. gamma) activity predominating in forward connections (prediction errors) and slower (e.g. beta) frequencies emerging as this evidence is accumulated in units that provide descending predictions [20–22].

At a more macroscopic level, the implicit anatomy of inference also provides a simple explanation for functional segregation [23]; in the sense that hierarchically deeper representations or expectations (e.g. what and where) are statistically segregated and are combined in a nonlinear way to contextualize lower-level causes of sensory information (e.g. colour and form). Indeed, one could argue that the very existence of slender axonal connections in the brain speaks to the sparse causal dependencies or laws that endow our sensory world with statistical regularities (contrast the anatomy of the brain with the anatomy of the liver). Although hierarchical predictive coding appears to have broad explanatory power, there is something missing from this picture. In short, there appears to be no role for corticothalamic interactions or (recursive) coupling with other subcortical structures. So, what is lacking in the above description of predictive coding?

### (b) Precision engineering and the encoding of context

One can regard ascending prediction errors as broadcasting ‘newsworthy’ information that has yet to be explained by descending predictions. However, the brain also has to select the channels it listens to—by adjusting the volume or *gain* of prediction errors that compete to update expectations in higher levels. Computationally, this gain corresponds to the precision or confidence associated with ascending prediction errors; very much in the same way that we assess the statistical significance of an effect in relation to its standard error. However, to optimally select the prediction errors—that should be afforded greater influence—the brain has to estimate or encode their precision. Having done this, prediction errors can then be weighted by their precision, so that only precise information is accumulated and assimilated in high or deep hierarchical levels.

This broadcasting of precision-weighted prediction errors may rest on neuromodulatory gain control mechanisms at a synaptic level [24]. This gain control corresponds to a (Bayes-optimal) encoding of precision in terms of the excitability of neuronal populations reporting prediction errors [2,19]. This may explain why superficial pyramidal cells have so many synaptic gain control mechanisms such as N-methyl-D-aspartate (NMDA) receptors and classical neuromodulatory receptors like D1 dopamine receptors [25–28]. Furthermore, it places excitation–inhibition balance in a prime position to mediate precision-engineered message passing within and among hierarchical levels [29]. As noted above, the encoding of salience or precision can also be understood in terms of synchronous gain [30] and fast (oscillatory) dynamics associated with binding, attention and dynamic coordination [4,5].

The dynamic and context-sensitive control of precision has been associated with attentional gain control in sensory processing [2,31] and has been discussed in terms of affordance and action selection [32–34]. Crucially, the delicate balance of precision over different hierarchical levels has a profound effect on inference—and may also offer a formal understanding of false inference in psychopathology [35]. To illustrate the potential importance of precision—and implicit synaptic gain control—we will look at a particular problem from the point of view of predictive coding and see why neuromodulatory mechanisms are an integral part of its solution.

## 3. Predictive coding and figure–ground segregation

This section considers the figure–ground segregation problem where, crucially, a figure is defined texturally—in terms of its second-order statistics; in other words, a visual object is manifest in terms of its texture or spectral power in the spatial domain. This segregation problem precludes recourse to first-order attributes, such as differences in luminance or colour. In other words, the quantities causing visual impressions are only defined in terms of their precision (or inverse variance). This presents an interesting problem for predictive coding (and the brain) that we use to illustrate the importance of gain control in finessing the inference problem.

In statistics, this (inverse) problem is usually solved using some form of variance component estimation; for example, using covariance constraints in the electromagnetic source reconstruction problem. Here, we solve the same problem with predictive coding. In this setting, hidden causes in the generative model control the precision or variance of subordinate causes generating data. Expectations of these hierarchical causes are optimized with respect to variational free energy—using predictive coding. Here, variational free energy is a proxy for Bayesian model evidence and can be regarded as the sum of the (squared and precision-weighted) prediction error. The simulation used to illustrate this solution is trivially simple but sufficient to make our key point, namely that top-down predictions have very different effects on prediction error responses—depending upon whether they encode the first- or second-order statistical properties of a stimulus.

We simulated a contiguous object, whose texture was determined by the variance of random fluctuations in luminance, where this variance was modulated by (Gaussian) spatial basis functions of retinotopic space. The resulting signal was mixed with uniform Gaussian observation noise to produce sensory data. These data were then subjected to Bayesian inversion using (generalized) predictive coding to recover the object or figure. The implicit figure–ground segregation basically involves estimating the hidden causes modulating the spatial basis functions controlling textural features—in this case, the local variance of stimulus intensity over sensory channels.

Technically, predictive coding optimizes expectations of the hidden causes of data that, in this case, include the amplitude of radial basis functions controlling the precision (inverse variance) of retinotopic signals (see below). In brief, we see that the resulting figure–ground segregation rests on selectively attending to sensory input from the figure, relative to the background. However, this form of attention is distinct from simply boosting sensory precision (the precision of sensory prediction errors) as in simulations of the Posner paradigm or biased competition [2]. Here, expectations of hidden causes are optimized in a way that renders them *less precise* and therefore more sensitive to ascending sensory (prediction error) input. This illustrates the importance of the *relative precision* of sensory and extrasensory prediction errors in modulating the influence of ascending sensory information (figure 2).

### (a) Simulation details

Three Gaussian basis functions 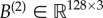 of a one-dimensional retinotopic space (with a separation and standard deviation of eight channels) were modulated with three hidden causes *v*^(2)^ = [8, 8, 0] to generate the log-precision of a visual signal over 128 visual channels. The resulting log precision vector 
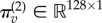
 was used to modulate Gaussian fluctuations 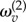 to generate textured signals by applying a Gaussian convolution matrix 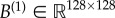 (with a standard deviation of two channels); finally, uniform Gaussian noise 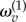 with a precision of 16 was added to the signals to generate sensory data3.1
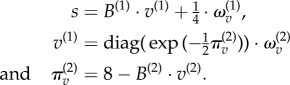


This way of generating data rests upon a generative model of the following form, which is a special case of the generative models described in the appendix: it is a special case, because there are no dynamics or hidden states3.2
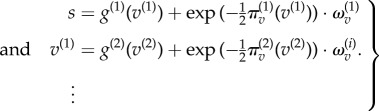


**Figure 3.**
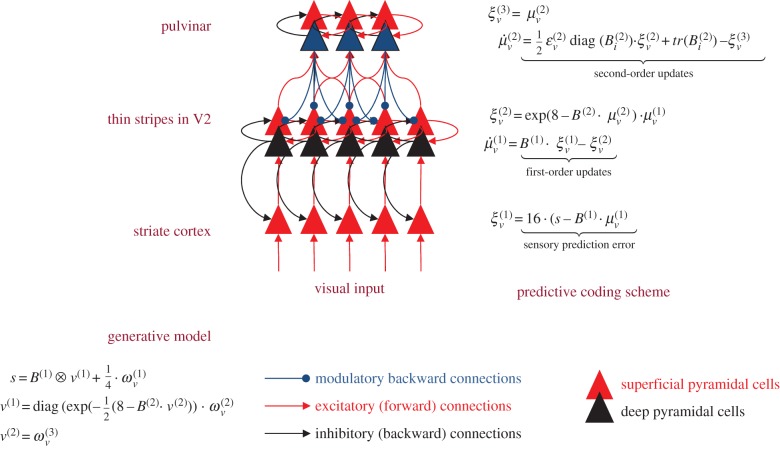
This schematic illustrates the message passing implicit in predictive coding based on the generative model described in the main text—and formulated mathematically on the lower left. In this scheme, sensory input is conveyed to visual cortex via ascending prediction errors from the lateral geniculate nucleus. Posterior expectations, encoded by the activity of deep pyramidal cells in primary visual cortex, are driven by ascending prediction errors while, at the same time, they are subjected to lateral interactions—with second-level prediction errors—that mediate (empirical) priors. Crucially, these constraints are modulated by top-down predictions of their precision (blue arrows). These predictions are based upon expectations about precision in the highest level that are effectively driven by the variance or power of prediction errors at the lower level. Heuristically, expectations about precision release posterior expectations from constraints in the vicinity of an inferred object and allow them to respond more sensitively to ascending geniculate input.

This generative model specifies the neuronal dynamics for posterior expectations about the hidden causes 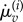 that constitute predictive coding (see appendix for details and figure 3 for the particular equations of the current model), where 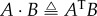:3.3
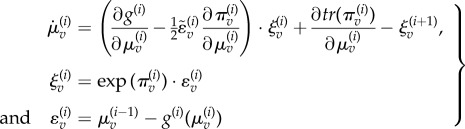

These equations provide a relatively simple update scheme, in which changes in posterior expectations are driven by a mixture of precision-weighted prediction errors—where prediction errors are defined by the equations of the generative model. Crucially, prediction errors are affected by descending predictions in one of two ways: expectations can either generate predictions of first-order effects, through the functions 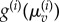. Alternatively, they can generate predictions of precision, through the functions 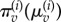. These effects are formally distinct: the first-order predictions (of lower expectations) have a negative (driving) effect on the prediction errors, whereas the second-order predictions (of their precision) have a positive (modulatory) effect. We can see this separation clearly in the current example, because the second-level hidden causes only predict second-order statistics (log precision), whereas the first-level hidden predict first-order statistics. This means equation (3.3) can be separated into first- and second-order updates3.4
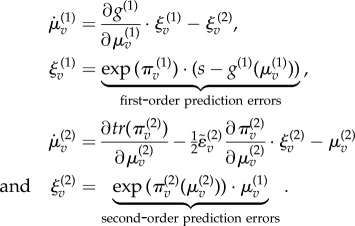


The key thing to take from these equations is the difference between first- and second-order message passing. The first-order expectations are driven by linear mixtures of first- and second-order prediction errors that play the role of the likelihood and (empirical) prior influences. Crucially, the second-order prediction errors (empirical priors) have more influence when they are more precise. Similarly, the first-order predictions enter the first-order prediction errors in a linear (subtractive) fashion. In contrast, the second-order expectations are driven by nonlinear (sum of squared) prediction errors and modulate the second-order prediction errors in a nonlinear fashion. It is this modulation we associate with precision-engineered message passing and the (attentional) contextualization of predictive coding. (See figure 3 for a schematic of this message passing for the simple model considered here.)

**Figure 4.**
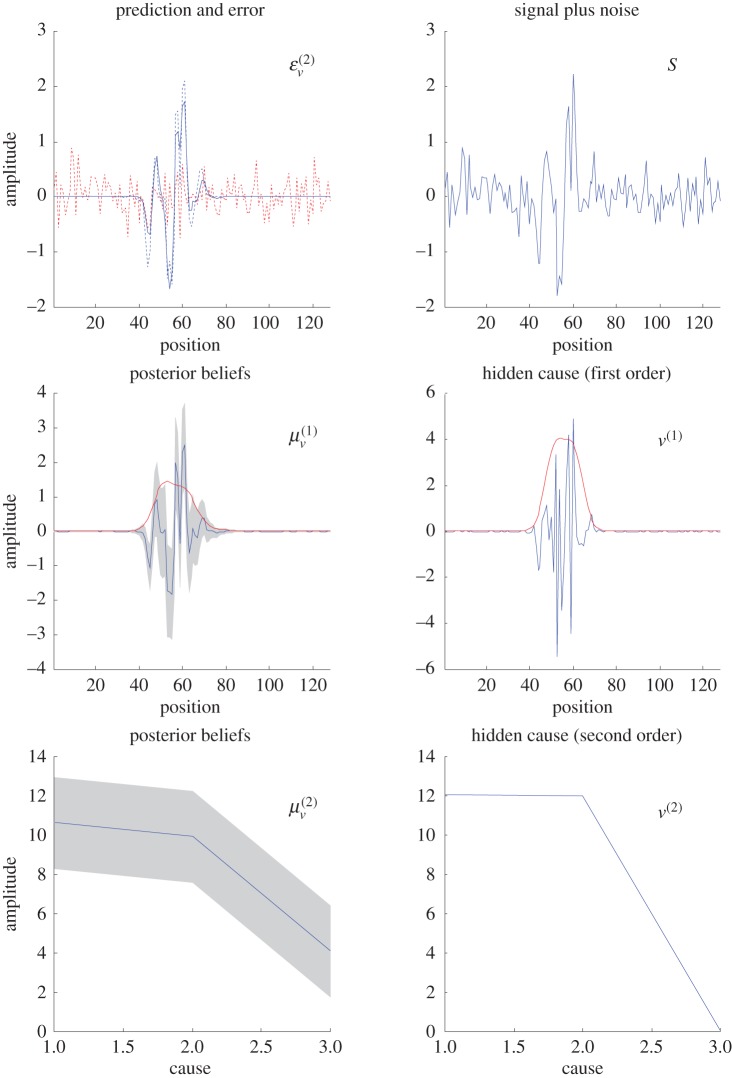
Results of generalized predictive coding. The key thing to take from these results is that the object has been segregated from the background, despite the high levels of sensory noise: predicted (solid blue line) and true (dotted blue line) sensory features are shown in the upper left panel with the associated prediction error (dotted red lines). Furthermore, the posterior confidence intervals (grey areas in the middle left panel) reveal the nature of precision-based predictive coding: note that the posterior confidence is reduced over the object or feature (whose location is shown on the middle right panel (red lines)—in terms of the expected standard deviation). This reduced confidence affords greater latitude for ascending sensory (prediction error) signals to influence posterior expectations. In contrast, over the radial basis functions in which signal was absent, the precision has increased, rendering these expectations insensitive to ascending prediction errors. This effectively means this (background) information is being ignored (or unattended).

Figure 4 shows the results of a typical simulation. The left columns show the results of predictive coding and the right columns show the true values generating sensory input. These inputs were inverted using the generative model that was used to produce them—but with unknown hidden causes at the first and second levels. The posterior expectations of these hidden causes are shown in the lower left panels—along with their 90% posterior confidence intervals (in grey). The upper left panel shows the predicted sensory input in blue, and the sensory prediction error in red.

**Figure 5.**
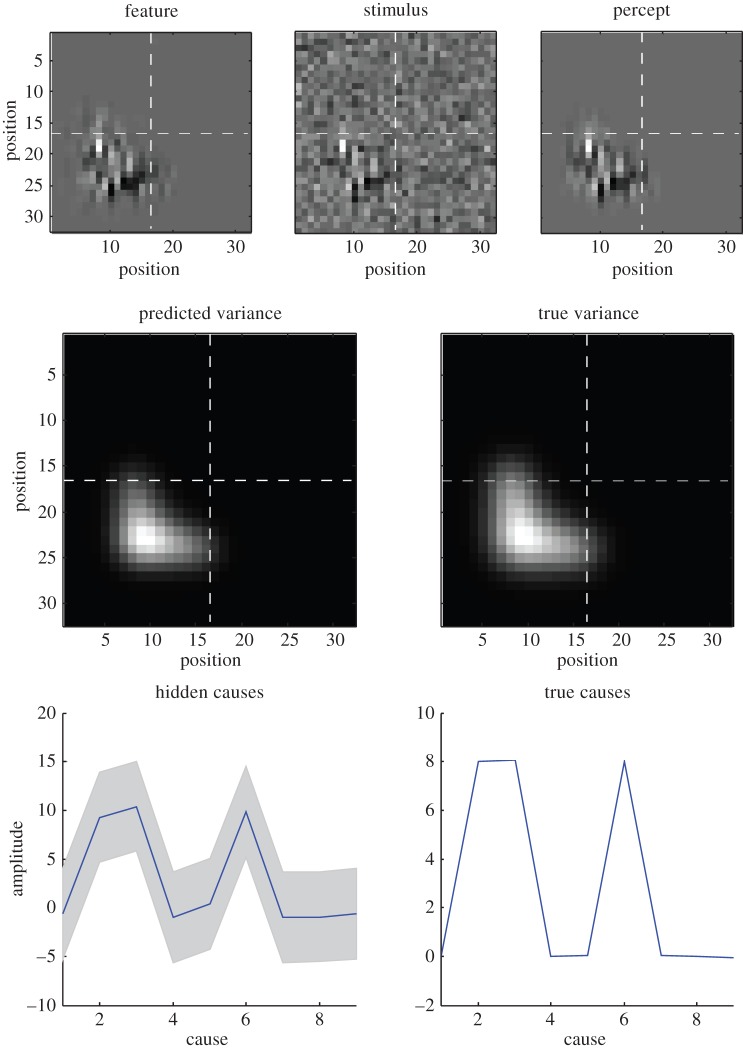
Generalized predictive coding in two dimensions. This shows the same sort of results as figure 4; however, here, the input has been formulated in two dimensions (over 32 × 32 visual channels). The upper row shows the true signal feature (left panel), the visual input after sensory noise has been added (middle panel) and the prediction based upon posterior expectations (right panel). It can be seen that the background noise has been suppressed by rendering expectations of local luminance very precisely. Conversely, the regime of signal enjoys more relaxed constraints and is effectively attended. This second-order effect is summarized in the middle row that shows the estimated (left panel) and true (right panel) profile of single variance (inverse precision), which—in this example—is a small L-shaped feature in the lower left quadrant. The associated (second level) hidden causes are shown in the lower row in terms of their posterior expectations and the true values (as in the previous figure). (Online version in colour.)

Figure 5 shows the results of the same simulation but using a two-dimensional visual input (and a grid of nine Gaussian radial basis functions). Here, the signal was an L-shaped feature (with anisotropic smoothness) in the lower left quadrant that has, effectively, attracted attention. This attentional ‘spotlight’ is nicely illustrated in terms of the expected variance (inverse precision) as shown in the middle panel. Although very simple, this example highlights the close relationship between attentional selection and figure–ground segregation based upon second-order statistics. Clearly, we could have used a much more elaborate generative model; for example, we could have considered anisotropic Gabor patches when mapping first level hidden causes to sensory (retinotopic) input (cf. classical receptive fields). One could also imagine having separate precision components for vertical and horizontal patches that themselves were constrained by higher hierarchical levels. We will illustrate these ideas in future papers. Here, we focus on the basic computational anatomy implied by these schemes.

### (b) Summary

These simulations demonstrate the nature of predictive coding, when sensory features generated by visual objects are textural or second-order in nature. They highlight the selective boosting or sensitivity to signals arising in the vicinity of a visual object or figure, suggesting a top-down augmentation of featural cues that is consistent with attentional selection. The abovementioned examples illustrate a form of precision or gain control that is intrinsic to the cortical hierarchy and speaks to separate descending streams of prediction—that predict the first and second-order attributes of lower-level representations. This scheme suggests that modulatory or gain control mechanisms are restricted to backward connections (the forward connections convey prediction errors, which are linear and driving). Note also that the descending predictions are effectively inhibitory, which is consistent with the targeting of inhibitory interneurons (particularly in layer one) by backward connections in the visual cortex [17]. In contrast, the predicted precision has an excitatory modulatory effect, consistent with mediation through voltage-dependent NMDA receptors in pyramidal cells of the superficial layers [19]. Later, we will also consider the important role of fast-spiking inhibitory interneurons and chandelier cells (that express NMDA receptors) in mediating synchronous gain.

Later, we consider general forms of descending precision control that have been associated with attentional processing. In this instance, the source of top-down gain control is not necessarily an intrinsic part of the cortical hierarchy but may call upon the cortical systems that control precision throughout the cortical hierarchy. So, what are the cardinal features a neuronal system should possess to mediate such precision control? Precision is a function of hidden causes, which means that expected precision depends on expected hidden causes that—like all expectations—we presume are encoded neuronally. A universal feature of predictive coding is that connections to populations encoding expectations are from populations encoding prediction errors, and these connections are reciprocated. In the special case of hidden causes of precision, these projections must show substantial (but possibly topographic) convergence and divergence: it can be seen from equation (3.4) (third equality) that the expected causes of precision gather information from each component or set of prediction errors that share the same covariance or precision. If the sum of (precision-weighted) squared prediction error is too large then expected precision falls and, conversely, rises when the sum of (precision-weighted) squared prediction error is too small. Furthermore, every prediction error unit contributing to the sum of squares receives reciprocal connections to modulate its gain or precision. Neuroanatomically, this suggests systems that encode and mediate expected precision must

— receive convergent projections from large (possibly topographically organized) regions of cortex, specifically from cells encoding prediction error (in supragranular layers);
— reciprocate divergent projections to the same regions;
— mediate some form of gain control over the cells encoding prediction error; and
— possess bilateral projections to cortical areas with corticocortical connections, to control the relative precision of their respective prediction errors.

In what follows, we consider corticothalamic systems—in which the thalamus (pulvinar) provides modulatory gain control—and what that implies for the cartography of attention and arousal.

## 4. Precision, attention and the pulvinar

There are two primary types of relay neurons in the thalamus, namely *core* cells and *matrix* cells [36]. Matrix cells are distributed widely over the nuclei of the dorsal thalamus and send thalamocortical axons that terminate principally in the superficial layers of the cortex [36,37]. Furthermore, thalamocortical signalling is primarily excitatory, but is largely mediated by inhibitory mechanisms that implement synchronous gain. Thalamocortical oscillations also provide modulatory inputs to the thalamus via GABAergic neurons that synapse in the reticular nucleus of the thalamus [38]. It seems natural therefore to consider (a subset of) the corticothalamic system as a candidate for precision control. In what follows, we review the evidence for such a role in the pulvinar—drawing on known neuroanatomy, neurophysiology and recent findings in cognitive neuroscience.

The pulvinar is the largest nucleus in the primate thalamus and has expanded in size during primate evolution—in parallel with other visual structures [39]. The pulvinar has long been thought to play a role in mediating visual attention [40,41] perhaps by registering the saliency of a visual scene [42,43]. Damage to the pulvinar in humans can result in visual hemi-neglect [44,45], deficits in feature binding [46] or focal attention [47]. Neurons in the pulvinar exhibit features of selective attention; as they respond more strongly to behaviourally relevant stimuli than to unattended stimuli [48], as such, they contribute to top-down suppression of distractors [49]. Human imaging studies report pulvinar activation that is consistent with the filtering of distractors [50–52], such that only information pertaining to the attended target can be decoded from patterns of activity [53].

Neuroanatomical observations of the pulvinar suggest that for every direct connection between two cortical regions, there is a parallel, indirect pathway that goes through the pulvinar. This is called the *replication principle* [41]. For example, consider the visual maps of ventral pulvinar [54,55] that receive retinotopically registered connections from the areas of the ventral visual pathway (V1, V2, V4, TEO and TE). These form a (diffuse) occipitotemporal gradient from V1 at one pole of the pulvinar map to area TE at the other. The relative overlap between the pulvinar fields of any given pair of areas roughly reflects their mutual level of cortical connectivity: for example, area V4 shares more pulvinar territory, and has stronger cortical connections with its neighbouring area TEO, than it does with the more distant area TE [41]. This neuroanatomical architecture of the cortico-pulvino-cortical pathway is therefore suited for concurrent precision estimation and to modulate the gain of reciprocal message passing between cortical hierarchical levels. In particular, the replication principle is entirely consistent with the control of the relative precision of prediction errors at different levels in the cortical hierarchy.

Based on these architectural properties—and the requirements of predictive coding—it seems reasonable to hypothesize that the functional role of the pulvinar is to optimize the gain of cortical prediction errors according to their expected precision. To fulfil this role, the pulvinar needs to encode expected precision and mediate gain modulation. Recent neurophysiological studies in behaving monkeys indicate that the pulvinar, indeed, performs these computational operations.

### (a) Empirical evidence for precision engineering in the pulvinar

In terms of encoding precision, it has been recently reported that approximately 30% of neurons in the pulvinar are sensitive to the reliability of task-relevant sensory signals, representing the ‘confidence’ in perceptual decisions [56]. During a perceptual decision task, this subset of neurons does not selectively respond to the content of perception, but exhibits a higher firing rate when the monkeys behaved as though they were certain about their perceptual decision. When the monkeys were given a choice to opt-out, for a smaller reward, a lower firing rate of these neurons predicted the escape response of the monkeys—even when the signal-to-noise ratio in the stimulus was identical. The firing rate was lower for more difficult trials, and the deactivation of these neurons by GABA agonist (muscimol) injection enhanced escape responses—as though the monkeys lost confidence in their perceptual decision even though their objective task performance was unimpaired. These findings support the notion that neurons in the pulvinar encode expected precision or confidence in information used for perceptual decisions.

The pulvinar's contribution to gain control has been demonstrated in a compelling study of spike-field coherence [57]. By concurrently recording pulvinar spikes and local field potentials from V4 and TEO, the authors showed that the spike-field coherence between the pulvinar neurons and alpha oscillation in V4 and TEO was enhanced when attention was directed to the receptive field of the pulvinar neuron. Crucially, conditional Granger causality analysis across the three regions showed that the pulvinar neurons facilitated the transmission of information between V4 and TE by synchronizing the alpha oscillation in those cortical regions. This provides empirical evidence that the pulvinar serves as a gain control system for corticocortical interaction—via controlling neuronal synchronization. This synchronous gain control offers a neurobiological mechanism to adjust effective synaptic gain transiently across cortical regions [55,58]. Furthermore, it is closely related to notions of communication through coherence (see below) and may reflect an important mechanism for precision engineering in attention [2].

These studies provide neurophysiological evidence that the pulvinar neurons encode expected precision, and modulate the gain of corticocortical communication. The notion of precision engineering in the pulvinar offers a coherent (computational) perspective on how seemingly disparate aspects of attention (gain modulation) and confidence (uncertainty estimation) are orchestrated. Although the concepts of salience, confidence and attention may appear distinct, their intimate relationship can be interpreted as an integral part of perceptual inference—reflecting the different faces of precision.

### (b) Gain control mechanisms in the cortico-pulvino-cortical connectivity

There are multiple thalamocortical mechanisms that can modulate the gain of prediction error in superficial layers of the cortex. Here, we consider three possible mechanisms through which the pulvinar contributes to gain control: (i) induction of phase synchrony across presynaptic neurons within a cortical region, perhaps achieved by (ii) modulation of low-frequency (alpha) oscillation across cortical regions and (iii) modulation of prediction error units in the superficial layers via diffuse projections.

The first mechanism reflects the fact that synchronized presynaptic spikes generally make the postsynaptic impact stronger. Thus, controlling the degree of synchrony at presynaptic neurons can serve as a gain control mechanism [59,60]. This can be achieved by synchronous modulation of subthreshold membrane potentials at the gamma frequency [61]. While gamma oscillations can be generated by intracortical mechanisms, the thalamus plays a role in modulating gamma oscillations in sensory cortex. For example, it has been shown that stimulation of the posterior intralaminar nucleus modulates synchronous gamma oscillations in the auditory cortex [62]. Extending this notion to the visual cortex, one may speculate that the pulvinar could modulate subthreshold gamma oscillation in prediction error neurons in the cortex, thereby controlling the synchrony of spike timings of prediction error neurons.

The second mechanism is phase synchronization of distant cortical areas by the pulvinar [41], invoking the principle of ‘communication through coherence’—that selective communication can be achieved through coherence between firing rate oscillation in the sending region and oscillatory gain modulation in the receiving region [63,64]. Corticothalamic connections play a prominent role in synchronizing oscillations [65], and the thalamus modulates phase relationships between cortical regions, thereby modulating the effective synaptic strengths. For example, Akam & Kullmann [66] demonstrate flexible signal routing in neural circuits, by exploiting sparsely synchronized network oscillations and temporal filtering by feed-forward inhibition.

The core cells in the pulvinar form a loop through layers 3 and 6 of extrastriate cortex [67]. This circuit could serve as an alpha generator for extrastriate cortex [68], through a mechanism analogous to the geniculocortical loop through layers 4 and 6 of V1 [69], and modulate the effectiveness of the output from one area to another distant cortical region. As discussed earlier, empirical evidence indicates that spikes from the pulvinar generate alpha rhythms in the target cortical areas, and induce corticocortical synchrony in the alpha frequency that facilitates communication between the synchronized cortical regions [57]. Furthermore, there is evidence in this study [57] and others [70,71] for alpha–gamma cross-frequency coupling, thus forging a link between the two mechanisms considered so far.

The third mechanism considers gain control by projections from the pulvinar to the superficial layers 1–3 of a visual area (e.g. V1). This diffuse projection, originating from the matrix cells of the pulvinar, can modulate the activity of prediction error neurons in the target area—a functional analogy with the superficial component of backward cortical connections that we have previously attributed with a role in precision control [19]. Gain control via this pathway has been empirically demonstrated. Inactivation of the lateral pulvinar suppressed responses of superficial V1 neurons to visual input, whereas excitation of the pulvinar neurons increased the responsiveness of neurons in the superficial layers with overlapping receptive fields [72]. Given the organization of cortico-pulvino-cortical connections (noted above), we predict that the pulvinar neurons sending feedback to superficial layers of any given cortical area receive information about expected precision via the descending input from areas at both higher and lower levels in the cortical hierarchy. This may differ from the corticocortical transmission of precision that we have so far linked exclusively to backward connections [19].

These candidate mechanisms suggest that the pulvinar has multiple ways to control the gain in corticocortical communications. These neuronal implementations of gain control mechanisms are by no means comprehensive, and are not mutually exclusive. While all of the mechanisms discussed here have some empirical support, which mechanism plays a dominant role in the context of the predictive coding framework remains to be determined. Nevertheless, these examples collectively point to the pulvinar's role in gain control in corticocortical communication.

### (c) Precision estimation in the corticothalamic network

**Figure 6.**
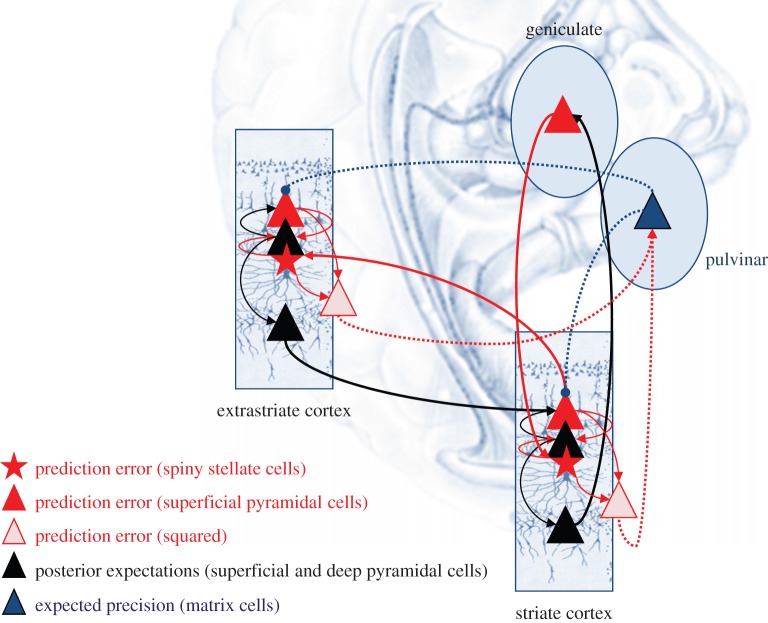
This schematic is a more detailed version of figure 2 that includes putative laminar-specific connections that are consistent with the precision-based predictive coding scheme in the main text. This architecture is based upon Bastos *et al.* [17] and Shipp *et al.* [19] and conforms roughly to the known neuroanatomy and physiology of canonical microcircuits and laminar specificity of extrinsic connections. The key aspect of this figure is the inclusion of deep pyramidal cells encoding the amplitude of prediction error (squared) that inform posterior expectations about precision in the (matrix cells) of the pulvinar. These cells reciprocate descending projections to modulate the gain of superficial pyramidal cells in cortex. Forward connections are in red and descending (backward) connections are in black. First-order streams are shown as full lines and second-order (precision-related) streams are shown as dashed lines.

The anatomy and laminar specificity of pulvinar projections to the cortex fits comfortably with the computational architecture implied by predictive coding (figure 6). We have outlined different roles for the core and matrix output neurons (in alpha generation for the core cells, projecting to the middle layers and in precision regulation for the matrix cells projecting superficially). The dual afferent projections to the pulvinar from the cortex derive from layers 5 and 6, thought to act as drivers and modulators, respectively [73,74]. It is these connections that should convey the (squared) prediction error to enable the pulvinar to estimate precision. However, if we consider the geniculocortical loop with striate cortex as a model for alpha generation [69] it is the layer 6 outputs to thalamus that serve this role, pointing to layer 5, perhaps, as the source of squared prediction error. Note that striate cortex output to pulvinar is not duplex, but arises exclusively from layer 5 [75]. All current analyses of canonical microcircuits place prediction error units in superficial layers [17–19]. Thus, we suppose that the principal cells reporting the squared prediction error (i.e. second-order forward connections) to the pulvinar are a secondary stream originating through the strong intrinsic connections from the superficial layers to layer 5. The particular arrangement that we arrive at—cortical drivers driving thalamic matrix cells, and cortical modulators modulating thalamic core cells—matches the anatomy reported for connections between frontal cortex and the ventral anterior thalamic nucleus [76], but the specificity of contacts existing within pulvinar is unknown.

The message passing implied by predictive coding would require these layer 5 principal cells to respond, in a U-shaped fashion, to both high and low levels of prediction error firing in superficial layers; in other words, be both excited and disinhibited by (first-order) prediction errors. The notion of a (second-order) forward-type corticopulvinar stream fits comfortably with the notion that input from layer 5 is largely feed-forward and the hypothesis that layer 5 corticothalamic axons represent the afferent limb of a corticothalamocortical pathway.

### (d) Summary

Here, the key requirements of a neuronal system that could coordinate precise corticocortical message passing among functioning segregated areas appears to be fulfilled by corticothalamic loops. A detailed consideration of the pulvinar, in relation to the computational anatomy of predictive coding, reveals a consistent picture at the architectural and microcircuit level—particularly with regard to the laminar specificity of intrinsic and extrinsic connections (and indeed suggests some new hypotheses about subpopulations and their connections). Furthermore, the emerging picture ties together a number of closely related themes, namely the distinction between driving and modulatory connections, cortical gain control, synchronous gain, communication through coherence and desynchronization of alpha rhythms. All of these physiological phenomena have been implicated in attentional processing and the encoding of salience or confidence, which we associate with precision control.

## 5. Conclusion

In this paper, we have considered how inferences about first-order content and second-order context are orchestrated in hierarchical predictive coding, highlighting the importance of modulatory effects by second-order representations—such as precision and saliency—in optimizing inference. We have considered the neurobiological substrates of precision engineering in the brain, with a special focus on the pulvinar and attention. In this proposal, inference about the (first-order) content of perception was ascribed to corticocortical message passing, whereas parallel corticothalamocortical connections contextualize (second-order) corticocortical processing via precision-weighted gain control of ascending prediction errors. This proposal offers a formal understanding of attentional functions and the encoding of expected precision by the pulvinar.

More generally, the notion of hierarchical inference in the brain provides a potentially important link between structure and function: if the brain transcribes causal structure from the world, then this (hierarchical) structure should be embodied in cortical architectures. Predictive coding provides a particular process theory for this transcription and calls for an understanding of microscopic (laminar-specific) message passing in canonical microcircuits—that is consistent with macroscopic cartography defined by extrinsic connections. The particular contribution of this paper is to highlight the context-sensitive and dynamic aspects of functional anatomy—distinguishing between the neuronal processing of (first-order) content and (second-order) context. The implications for the future of cerebral cartography are manifest at a number of levels, namely a fuller understanding of the asymmetries between forward and backward connections—and the distinction between streams responsible for perceptual synthesis *per se* and those (presumably more diffuse) streams that contextualize perceptual processing, enabling the selection and coordination of precise information. The formal constraints offered by schemes like predictive coding highlight the need to characterize cortical interactions at the level of cortical layers and the orchestration of cerebral processing through centrifugal exchanges with subcortical structures.

## Appendix A

This brief description of generalized predictive coding is based on Feldman & Friston [2]. A more technical description can be found in Friston *et al.* [77]. This scheme is based on three assumptions

— The brain minimizes a free energy of sensory inputs defined by a generative model.
— The generative model used by the brain is hierarchical, nonlinear and dynamic.
— Neuronal firing rates encode the expected state of the world under this model.

Free energy is a quantity from statistics that measures the quality of a model in terms of the probability that it could have generated observed outcomes. This means that minimizing free energy maximizes the Bayesian evidence for the generative model. The second assumption is motivated by noting that the world is both dynamic and nonlinear, and that hierarchical causal structure emerges inevitably from a separation of spatial and temporal scales. The final assumption is the Laplace assumption that leads to the simplest and most flexible of all neural codes.

Given these assumptions, one can simulate a whole variety of neuronal processes by specifying the particular equations that constitute the brain's generative model. In brief, these simulations use differential equations that minimize the free energy of sensory input using a generalized gradient descent.

A 1

These differential equations say that neuronal activity encoding posterior expectations about (generalized) hidden states of the world  reduce free energy—where free energy  is a function of sensory inputs  and neuronal activity. This is known as generalized predictive coding or Bayesian filtering. The first term is a prediction based upon a differential matrix operator *D* that returns the generalized motion of expected hidden states . The second (correction) term is usually expressed as a mixture of prediction errors that ensures the changes in posterior expectations are Bayes-optimal predictions about hidden states of the world. To perform neuronal simulations under this scheme, it is only necessary to integrate or solve equation (A 1) to simulate the neuronal dynamics that encode posterior expectations. Posterior expectations depend upon the brain's generative model of the world, which we assume has the following hierarchical form:

A 2

Equation (A 2) describes a probability density over the sensory and hidden states that generate sensory input. Here, the hidden states have been divided into hidden states and causes (*x*^(*i*)^, *v*^(*i*)^) at the *i*th level within the hierarchical model. Hidden states and causes are abstract variables that the brain uses to explain or predict sensations—like the motion of an object in the field of view.

In these models, hidden causes link hierarchical levels, whereas hidden states link dynamics over time. Here, (*f*^(*i*)^, *g*^(*i*)^) are nonlinear functions of hidden states and causes that generate hidden causes for the level below and—at the lowest level—sensory inputs. Random fluctuations in the motion of hidden states and causes  enter each level of the hierarchy. Gaussian assumptions about these random fluctuations make the model probabilistic. They play the role of sensory noise at the first level and induce uncertainty at higher levels. The amplitudes of these random fluctuations are quantified by their precisions that may depend upon the hidden states or causes through their log-precisions .

Given the form of the generative model (equation (3.2)) we can now write down the differential equations (equation (A 1)) describing neuronal dynamics in terms of (precision-weighted) prediction errors. These errors represent the difference between posterior expectations and predicted values, under the generative model (using  and omitting higher-order terms):

A 3

This produces a relatively simple update scheme, in which posterior expectations  are driven by a mixture of prediction errors  that are defined by the equations of the generative model.

In neural network terms, equation (A 3) says that error-units compute the difference between expectations at one level and predictions from the level above (where  are precision-weighted prediction errors at the *i*th level of the hierarchy). Conversely, posterior expectations are driven by prediction errors from the same level and the level below. These constitute bottom-up and lateral messages that drive posterior expectations towards a better prediction to reduce the prediction error in the level below. In neurobiological implementations of this scheme, the sources of bottom-up prediction errors are generally thought to be superficial pyramidal cells, because they send forward (ascending) connections to higher cortical areas. Conversely, predictions are thought to be conveyed from deep pyramidal cells by backward (descending) connections, to target the superficial pyramidal cells encoding prediction error [16,17].

Note that the precisions depend on the expected hidden causes and states. We have proposed that this dependency mediates attention [2]. Equation (A 3) tells us that the (state-dependent) precisions modulate the responses of prediction error units to their presynaptic inputs. This suggests something intuitive—attention is mediated by activity-dependent modulation of the synaptic gain of principal cells that convey sensory information (prediction error) from one cortical level to the next. This translates into a top-down control of synaptic gain in principal (superficial pyramidal) cells and fits comfortably with the modulatory effects of top-down connections in cortical hierarchies that have been associated with attention and action selection.
